# The Ubiquity of the Reaction of the Labile Iron Pool That Attenuates Peroxynitrite-Dependent Oxidation Intracellularly

**DOI:** 10.3390/biom14070871

**Published:** 2024-07-19

**Authors:** Gabriel Simonetti da Silva, Maria Beatriz Braghetto Hernandes, José Carlos Toledo Junior

**Affiliations:** Departamento de Química, Faculdade de Filosofia, Ciências e Letras de Ribeirão Preto, Universidade de São Paulo, Ribeirão Preto 14040-901, SP, Brazil

**Keywords:** labile iron pool, labile iron, LIP, chelatable iron pool, CIP, iron, peroxynitrite, nitric oxide, superoxide

## Abstract

Although the labile iron pool (LIP) biochemical identity remains a topic of debate, it serves as a universal homeostatically regulated and essential cellular iron source. The LIP plays crucial cellular roles, being the source of iron that is loaded into nascent apo-iron proteins, a process akin to protein post-translational modification, and implicated in the programmed cell death mechanism known as ferroptosis. The LIP is also recognized for its reactivity with chelators, nitric oxide, and peroxides. Our recent investigations in a macrophage cell line revealed a reaction of the LIP with the oxidant peroxynitrite. In contrast to the LIP’s pro-oxidant interaction with hydrogen peroxide, this reaction is rapid and attenuates the peroxynitrite oxidative impact. In this study, we demonstrate the existence and antioxidant characteristic of the LIP and peroxynitrite reaction in various cell types. Beyond its potential role as a ubiquitous complementary or substitute protection system against peroxynitrite for cells, the LIP and peroxynitrite reaction may influence cellular iron homeostasis and ferroptosis by changing the LIP redox state and LIP binding properties and reactivity.

## 1. Introduction

The identity of the ubiquitous cellular iron reservoir, known as the labile iron pool (LIP), remains inadequately characterized. The current methodological definition describes the LIP as a cellular iron fraction that can be displaced from its cellular ligands by high-affinity LIP chelators [1]. This methodological definition, however, is overly broad, connecting iron sources solely based on their chelatable property, and is commonly used to describe distinct redox-active iron that develops in various unrelated and dysregulated conditions [2,3]. It is crucial to emphasize that under normal physiological conditions, the LIP represents a universal iron pool [4,5,6]. Its ferrous redox state is maintained and its concentration is regulated by canonical iron homeostasis mechanisms, with positive or negative perturbations met with complementary homeostatic actions that restore the LIP status [7,8,9,10,11,12,13,14,15]. Moreover, the LIP plays fundamental roles within cells. Notably, the LIP is loaded into newly synthesized apo-iron proteins [16,17,18], a process akin to protein post-translational modification, and appears to be increased and involved in the programmed cell death mechanism known as ferroptosis [19]. Also, our recent findings demonstrate that the LIP binds with high affinity (Kd in the order of 10^−2^ µM) to cellular constituents found in low concentrations (sub to low µM) and exhibits remarkably similar binding properties across different cell types [20]. While the molecular nature of these constituents serving as LIP ligands remains unknown, these observations advance our understanding of the LIP and suggest that it has similar molecular chemical identities across various cells.

**Scheme 1:**
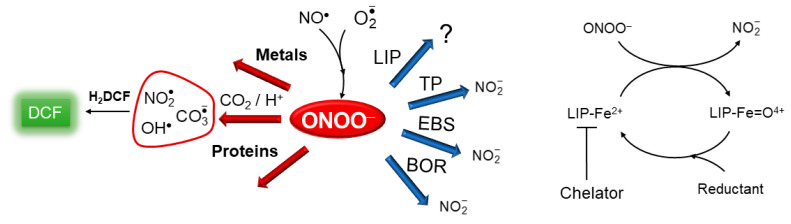
The peroxynitrite reactivity and its hypothetical reaction with the LIP. Reactivity of peroxynitrite with endogenous and exogenous cellular targets (**left**): thiol peroxidases (TP), metals, proteins, CO_2_ (phenomenologically producing 2/3 of oxidant radicals), and possibly the LIP. Exogenous chemicals such as Ebselen (EBS) and Boronates (BOR) reduce peroxynitrite to nitrite (${\mathrm{NO}}_{2}^{-}$). Hypothesis (**right**): The ferrous LIP directly reacts with peroxynitrite, presumably producing ${\mathrm{NO}}_{2}^{-}$. Such reaction is inhibited by LIP chelation, increasing the availability of peroxynitrite and the net yield of its derived radicals, which ultimately enhance the peroxynitrite-dependent direct and indirect oxidation of endogenous and exogenous (H_2_DCF) cellular targets.

The reactivity of the LIP has been overlooked, with a focus primarily on LIP chelation and its assumed Fenton-like reaction with hydrogen peroxide (H_2_O_2_) that generates powerful oxidants. Quite possibly, however, the most important aspect of LIP reactivity involves its interaction with nitrogen monoxide (nitric oxide, NO^•^) [21,22,23]. This reaction quantitatively yields dinitrosyl iron complexes (DNIC) [21] and holds the potential to impact the biological availability and functions of both NO^•^ and the LIP. This encompasses the classic regulatory functions of NO^•^, such as vasodilation, neurotransmission, protein nitrosylation [24,25], and cellular iron homeostasis [9,10,21] and ferroptosis [26]. Using a model of macrophage cells (RAW 264.7 cells), our investigations have revealed that the LIP also reacts with peroxynitrite (Scheme 1) [27,28], a reactive oxidant formed by the diffusion-limited radical recombination reaction of NO^•^and superoxide ($O_{2}^{\bar{•}}$) [29]. Upon protonation or reaction with CO_2_, peroxynitrite ultimately generates aggressive species, such as hydroxyl (OH^•^) [30,31], nitrogen dioxide (${\mathrm{NO}}_{2}^{•}$), and carbonate anion ($CO_{3}^{\bar{•}}$) [32] radicals (Scheme 1), which have the potential to oxidize biological molecules.

Notably, in contrast to LIP and H_2_O_2_-induced oxidation, in cells exposed to concurrent fluxes of both NO^•^ and $O_{2}^{\bar{•}}$, the LIP consistently attenuates peroxynitrite-dependent oxidation and nitrosylation of intracellular indicators under simulated normal and increasing oxidative conditions [27,28]. Despite the neglect of this reaction, it is anticipated, as transition metals are preferential targets of peroxynitrite [33,34], and as virtually all chemical species that react with H_2_O_2_ also react with peroxynitrite, typically at higher rate constants. We hypothesized (Scheme 1) that the LIP reduces peroxynitrite to ${\mathrm{NO}}_{2}^{-}$. Based on our findings, the LIP and peroxynitrite reaction is kinetically competitive with other potential peroxynitrite targets [28] and antioxidant enzymes. These findings underscore the significance of considering the diverse reactivity of the LIP, especially its interaction with NO^•^ and peroxynitrite, in understanding cellular redox dynamics. In this study, we present evidence indicating that the reaction between the LIP and peroxynitrite is widespread and exhibits consistent properties across different cell lines.

## 2. Materials and Methods

### 2.1. Materials

Unless otherwise specified, all chemicals were sourced from Sigma-Aldrich (St. Louis, MO, USA) and met the highest available purity standards. The chelator salycylaldehyde isonicotinoyl hydrazone (SIH) was synthesized following the procedure described previously [35] (^1^H NMR (400 MHz, DMSO-d6) δ (ppm): 12.33 (s, 1H), 11.11 (s, 1H), 8.81 (dd, J = 4.5, 1.5 Hz, 2H), 8.69 (s, 1H), 7.86 (dd, J = 4.5, 1.5 Hz, 2H), 7.62 (dd, J = 7.7, 1.5 Hz, 1H), 7.38–7.28 (m, 1H), 7.00–6.90 (m, 2H); ^13^C NMR (100 MHz, DMSO-d6) δ (ppm): 161.35, 157.43, 150.33, 149.01, 139.97, 131.73, 129.21, 121.49, 119.43, 118.63, 116.41). The SIH purity was verified to be higher than 95%. NO^•^ donors were obtained from Cayman Chemical Co (Ann Arbor, MI, USA), and Calcein-AM and Calcein (CA) were sourced from Biotium (Fremont, CA, USA). All stock solutions, including NO^•^ donors, 2-phenyl-1,2-benzoselenazol-3-one (EBS), sodium hexacyanoferrate (II) (FCN), and SIH, were prepared, maintained, and quantified as previously established [27,28].

### 2.2. Cell Culture and Treatment

Similar to RAW 264.7 cells [27,28], HEPG2, HEK293, and U87MG cells (ATCC) were incubated and cultured at 37 °C in Dulbecco’s Modified Eagle’s Medium supplemented with 100 units/mL penicillin, 100 µg/mL streptomycin-penicillin, and 10% fetal bovine serum (FBS). Cells were passaged, seeded onto 75 cm^2^ T-flask culture dishes, and allowed to grow overnight to achieve 85 to 90% confluence. Subsequently, the cells were double-washed with PBS, harvested, and centrifuged at 450× *g* for 5 min at 4 °C. The cells were then subjected to different treatments as described below. The trypan blue exclusion assay was conducted both before and after selected experiments to ensure that cell viability remained above 85%.

### 2.3. Loading of Indicator Procedures and Fluorescence Experiments

Suspensions of various cell types in PBS supplemented with 100 μM diethylenetriaminepentaacetic acid (DTPA) (PBS/DTPA) were exposed to peroxynitrite containing 10 μM of coumarin-7-boronic acid (CBA) (12 × 10^6^ cell/mL), or loaded with 30 μM 2′,7′-dichlorodihydrofluorescein diacetate (H_2_DCF-DA) (60 × 10^6^ cell/mL) for 30 min or varying concentrations of Calcein-AM (45 × 10^6^ cell/mL) for 20 min under continuous stirring at 37 °C. To minimize differences in the intracellular concentrations of fluorescent indicators during experiments, an identical number of cells was consistently used in the probe loading procedures. Once the probes permeated biological membranes, the ester bonds of H_2_DCF-DA and Calcein-AM were cleaved by nonspecific esterases, and the respective products H_2_DCF and CA were trapped and accumulated intracellularly. To eliminate extracellular H_2_DCF and CA, cells underwent cycles of centrifugation and resuspension in probe-free buffer following the loading procedure, with another cycle just before the experiment. Fluorescence measurements were taken at designated time intervals indicated in the figures.

### 2.4. Quantification of LIP

The cytosolic LIP concentration in cells was assessed using a modified CA assay [36]. In brief, a suspension of 50 × 10^6^ cells in 3 mL of PBS was loaded with Calcein-AM (0.25, 0.5, 1.0, 2.0, and 3.0 µM) and manipulated as described above to eliminate extracellular CA [36]. Then, a suspension of CA-loaded cells in pre-warmed (37 °C) PBS/DTPA (15 × 10^6^ cells in a total volume of 3.0 mL, with 90–95% viability) was placed in a conventional fluorimeter (Shimadzu RF-5301pc spectrofluorometer) in a quartz cuvette under constant stirring and controlled temperature. After establishing a fluorescence baseline, the LIP chelator SIH was added, leading to an increase in fluorescence. The fluorescence before the addition of the chelator was proportional to the free CA, and the difference between the initial and final fluorescence after SIH addition was proportional to the LIP-bound CA (CALIP). The concentrations of free CA and CALIP in the bulk solution were determined using a standard analytical curve of fluorescence versus free CA, generated by successive additions of known concentrations of free CA to a suspension of control cells in the presence of SIH [36]. The CA stock solution was prepared in DMSO, and its concentration was determined using absorbance at 492 nm and the molar absorptivity coefficient of ε492 = 7.5 × 10^4^ M^−1^ cm^−1^. The measurements were performed with the following fluorescence acquisition parameter settings: λ excitation = 495 nm, λ emission = 516 nm, and an excitation and emission slit width of 3 nm. Intracellular free CA and CALIP concentrations were calculated by molar equivalence using the following equation: solution [CA] or solution [CALIP]) × assay total volume/total volume of cells [20]. For the calculation of the total volume of cells, the cell diameter was obtained from the literature (Sizes of various cells.pdf; https://bionumbers.hms.harvard.edu/ (accessed on 23 June 2022)). The total LIP concentration (LIP_T_) was determined by fitting plots of paired CALIP × CA to a hyperbolic equation using Origin 2022 software (OriginLab Corporation, Northampton, MA, USA) as described elsewhere [28].

### 2.5. Generation and Detection of Peroxynitrite

The flux of peroxynitrite was achieved by co-producing $O_{2}^{\bar{•}}$ and NO^•^. The NO^•^ source in these experiments was the donor 2,2′-(Hydroxynitrosohydrazono)bis-ethanimine (DETA/NO), which has a half-life of 20 h at 37 °C and pH 7.4, respectively [37]. The $O_{2}^{\bar{•}}$ flux was generated by the use of 2,3-dimethoxy-1,4-naphthalenedione (DMNQ) [24], which catalytically generates intracellular $O_{2}^{\bar{•}}$ at the expense of cellular reducing agents [38]. We measured the NO^•^ concentration amperometrically in a cell suspension using an NO^•^-selective electrode in the absence and presence of the LIP chelator SIH. The NO^•^ steady state concentration, which ranged within 100 to 150 nM in the absence of DMNQ, was not affected by SIH, similar to that observed in RAW 264.7 cells [27]. All experiments were conducted in PBS/DTPA, and the temperature was maintained at 37 °C. The formation of peroxynitrite was monitored fluorometrically using CBA. Cell suspensions containing 10 µM CBA were transferred to 96-well plates (3 × 10^6^ cells per pool in 250 μL) in the presence of DMNQ and DETA/NO, and experiments were conducted using a plate reader (SpectraMax i3x, Molecular Device, San Jose, USA) with the following fluorescence acquisition parameter settings: λex = 332 nm, λem = 456 nm, and ex and em slit width = 9 nm and 15 nm, respectively.

### 2.6. Peroxynitrite-Dependent Oxidation of H_2_DCF in the Absence and Presence of an LIP Chelator

The flux of peroxynitrite in cells was achieved as described above. Suspensions of H_2_DCF pre-loaded cells were transferred to 96-well plates (3 × 10^6^ cells per pool in 250 μL) in the presence of DMNQ or DETA/NO alone or in the presence of both DMNQ and DETA/NO ± SIH (100 μM). The experiments were conducted using a plate reader (SpectraMax i3x, Molecular Device, San Jose, USA) with the following fluorescence acquisition parameter settings: λ excitation = 498 nm and λ emission = 523 nm, with excitation and emission slit widths set at 9 nm and 15 nm, respectively. 

### 2.7. Statistical Analysis

All measurements are presented as the means ± S.D. of n ≥ 4 experiments. Means were compared between groups using an F-test followed by Student’s *t*-test, employing the academic version of Origin 2022 software (OriginLab Corporation, Northampton, MA, USA). *P* values of < 0.05 were considered statistically significant.

## 3. Results

### 3.1. The Cytosolic LIP Concentration in Cells

**Figure 1:**
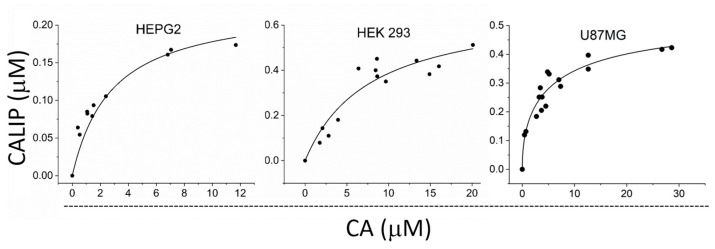
Plots of paired intracellular concentrations of CALIP and CA in different cell types. The LIP_T_ concentration was determined by computer adjustment as the limiting CALIP concentration by fitting the data to a hyperbolic equation using Origin 2022 software_._ The LIP_T_ concentration was as follows: HEPG2, 0.26 ± 0.03 µM; HEK 293, 0.7 ± 0.1 µM; and U87-MG, 0.53 ± 0.03 µM. Reported deviations are asymptotic standard errors provided by Origin. Part of our own data were reproduced with the permission of Springer Nature and Copyright Clearance Center (Insights on the endogenous labile iron pool binding properties|BioMetals (springer.com (accessed on 23 June 2024)) [20].

First, we quantified the cytosolic LIP content in the different cell types selected for the study using a modified CA fluorescence methodology, as described previously [28]. CA binds the cellular LIP stoichiometrically to produce the CALIP complex [36]. We loaded cells with different concentrations of CA and determined paired CALIP and CA intracellular concentrations. The plots of CALIP as a function of increasing CA concentrations expectedly resembled a hyperbolic binding curve reaching a plateau (Figure 1). The LIP_T_ concentration was determined by computer adjustment as the limiting CALIP concentration, as described elsewhere [28]. The best fittings for each cell type of the study are shown in Figure 1. The cytosolic LIP_T_ concentrations were as follows: HEPG2, 0.26 ± 0.03 µM; HEK 293, 0.7 ± 0.1 µM; and U87-MG, 0.53 ± 0.03 µM. For RAW264.7 cells, the LIP_T_ concentration was estimated previously to be 1.9 ± 0.4 µM [28].

### 3.2. Generation of Peroxynitrite Fluxes in Cells

**Figure 2:**
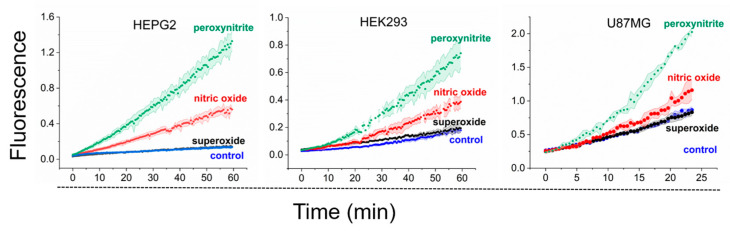
Formation of peroxynitrite by DMNQ/NO^•^ in cells. COH fluorescence traces of HEPG2 (**left**), HEK 293 (**center**), and U87-MG (**right**) cells exposed to different species. Control (black): no treatment; Superoxide (blue): DMNQ (2 µM); Nitric Oxide (red): DETA/NO (250 µM); Peroxynitrite (green): DMNQ (2 µM) and DETA/NO (250 µM). Briefly, cell suspensions containing CBA (10 µM) were transferred to 96 well plates (12 × 10^6^ cells/mL). DMNQ (2 µM) and DETA/NO (250 µM) were individually added to selected wells in this order. Fluorescence acquisition was registered every minute for at least one hour. The signal of U87MG cells saturated after 25 min. Data represent the mean of four independent experiments ± S.D. The experiments were performed in PBS containing DTPA (100 µM) at pH 7.4 and 37 °C using a multiple well plate reader with the following fluorescence acquisition parameters: λex = 332 m, λem = 456 nm, and ex and em slit width = 9 nm and 15 nm, respectively.

Before addressing the existence of the reaction between the LIP and peroxynitrite, we established experimental conditions for peroxynitrite generation in different cell types in suspension. This was achieved by co-producing $O_{2}^{\bar{•}}$ and NO^•^ with DMNQ and DETA/NO, respectively, a strategy successfully employed in RAW 264.7 cells [27,28]. The combination of DMNQ and DETA/NO is referred to as DMNQ/NO^•^. Peroxynitrite production in cell suspensions was monitored using 10 µM CBA through fluorescence spectroscopy. CBA reacts with peroxynitrite with a high rate constant (*k* = 1.1 × 10^6^ M^−1^s^−1^) [39], yielding the fluorescent product 7-hydroxy coumarin (COH) (Figure 2). The oxidation of CBA in the presence of DMNQ but in the absence of NO^•^ (labeled superoxide; Figure 2) did not differ from that of the control. In the presence of NO^•^ alone (labeled nitric oxide; Figure 2), CBA oxidation increased, likely due to the peroxynitrite generated through the reaction of NO^•^ and endogenous $O_{2}^{\bar{•}}$ produced by respiring cells. With DMNQ/NO^•^ treatment (labeled peroxynitrite; Figure 2), the oxidation further increased, indicating successful peroxynitrite generation in all selected cell types.

### 3.3. LIP Removal by Chelation Increases Peroxynitrite-Dependent Intracellular Oxidation of a Fluorescent Indicator in Cells

**Figure 3:**
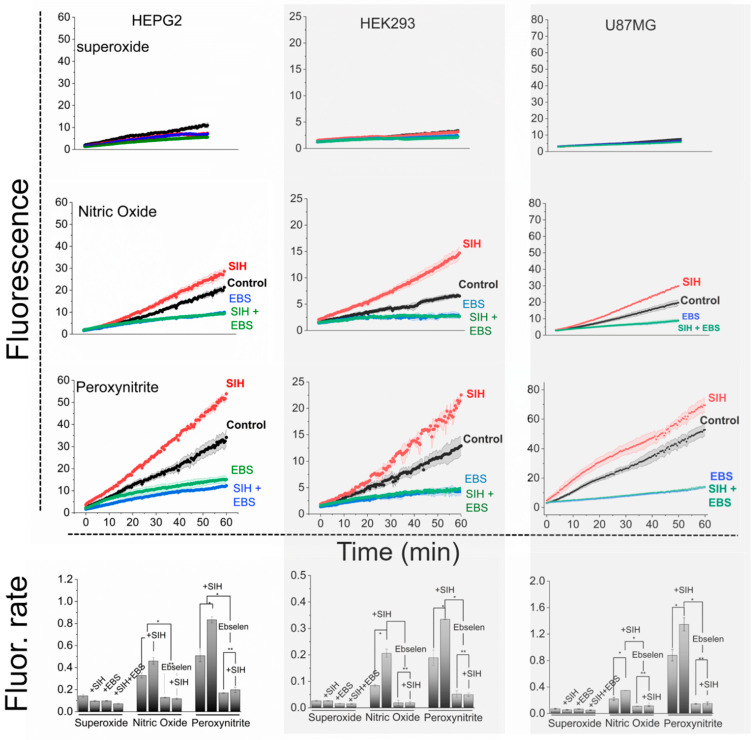
DCF fluorescence traces and rate of DCF fluorescence increase in HEPG2, HEK 293, and U87MG cells exposed to fluxes of different species in the absence and in the presence of SIH. Left column: HEPG2 cells; center column: HEK 293 cells; right column: U87MG cells. (**Top row**): superoxide-DMNQ (2 µM); second row: Nitric Oxide-DETA/NO (250 µM); third row: peroxynitrite-DMNQ (2 µM) and DETA/NO (250 µM). Control (black traces): cells treated with DMNQ (**top row**), DETA/NO (**second row**), or DMNQ and DETA/NO (**third row**); SIH (red traces): control + SIH (100 µM); EBS (green traces): control + EBS (50 µM); SIH + EBS (blue traces): control + SIH (100 µM) + EBS (50 µM). (**Bottom row**): the rate of DCF fluorescence increase in cells treated with different species. This rate was determined by linear regression of the fluorescence traces presented in the respective panels of each cell type using the average data collected during the final 10 min of each run. Data represent the mean of four independent experiments ± S.D. and are statistically significant at the 95% confidence interval relative to control cells (* *p* < 0.05). The Ebselen ± SIH groups are not statistically significant (**p > 0.05—not significant). Briefly, H_2_DCF-loaded cells were transferred to 96-well plates (12 × 10^6^ cells/mL). DMNQ, SIH, EBS, and DETA/NO were individually added to selected wells in this order. Fluorescence data acquisition was initiated immediately after the introduction of DETA/NO and registered every minute for one hour (**three top rows**). The experiments were performed in PBS containing DTPA (100 µM) at pH 7.4 and 37 °C using a multiple well plate reader with the following fluorescence acquisition parameters: λex = 498 nm, λem = 523 nm, and ex and em slit width = 9 nm and 15 nm, respectively.

Next, we replicated the critical experiments previously performed in RAW 264.7 cells using suspensions of cells of various types to investigate the existence and consequences of the hypothetical reaction between the LIP and peroxynitrite (Figure 3). As depicted in Scheme 1, CO_2_, transition metals, proteins, thiol peroxidases (TPs) such as glutathione peroxidases [40] and peroxiredoxins [41], and the LIP compete for peroxynitrite in cells. CO_2_ reacts rapidly with peroxynitrite and has been shown to compete for it with other cellular targets [42,43]. Investigation of LIP properties and reactivity is challenging due to the unknown identity of the LIP. Typically, we compare data obtained in cells under identical conditions, both in the absence and presence of a membrane-permeable LIP chelator. Thus, by employing specific chelators, the influence of the LIP on oxidative processes can be studied. In cells exposed to peroxynitrite fluxes, the presence of LIP chelators led to an increase in the oxidation of intracellular fluorescent indicators [27,28]. In our studies, we selected the LIP chelator SIH, grounded on several key properties (please see the Appendix A for more details).

Consistent with our previous studies [27,28], we employed H_2_DCF for monitoring peroxynitrite-dependent oxidation in cell suspensions through fluorescence spectroscopy. Although H_2_DCF does not directly react with peroxynitrite, it exhibits high rate constants with all peroxynitrite-derived radical oxidants (OH^•^, ${\mathrm{NO}}_{2}^{•}$, and ${\mathrm{CO}}_{3}^{\bar{•}}$) [44,45] (Scheme 1), ultimately leading to the formation of the oxidized and highly fluorescent product dichlorofluorescein (DCF). Concerns about artefactual production of $O_{2}^{\bar{•}}$ by H_2_DCF were addressed by deliberately generating $O_{2}^{\bar{•}}$ with DMNQ, ensuring that H_2_DCF-derived $O_{2}^{\bar{•}}$ was negligible under the experimental conditions of the study. Further details addressing criticisms of using H_2_DCF are available in Appendix B. 

The cells previously loaded with H_2_DCF were exposed to DMNQ (labeled superoxide), DETA/NO (labeled nitric oxide), and DMNQ/NO^•^ (labeled peroxynitrite) in 96-well plates, as detailed earlier [28]. The fluorescence of DCF was monitored in real time (Figure 3; three top panels in each column) and highlighted in the data compiled in the bottom panels of Figure 3, which show the rate of H_2_DCF oxidation. To facilitate a direct comparison of indicator oxidation by the different treatments, we expressed the data of the different species for each cell type on the same scale.

The $O_{2}^{\bar{•}}$ flux alone had no impact to a minimal impact on H_2_DCF oxidation under our experimental conditions, being statistically insignificant relative to the control, and was unaffected by EBS or SIH (Figure 3, top panels). In the presence of NO^•^ and peroxynitrite, the oxidation of H_2_DCF was significant and proportional to peroxynitrite production in the different cells (compare Figure 2 and Figure 3 data), increasing sequentially from NO^•^ to peroxynitrite fluxes. In striking contrast to $O_{2}^{\bar{•}}$, the oxidation of H_2_DCF in the presence of NO^•^ and peroxynitrite was inhibited in the presence of EBS and enhanced in the presence of SIH (Figure 3). The divergent effects of EBS and SIH highlight fundamental distinctions in the mechanisms and the role of SIH between $O_{2}^{\bar{•}}$ (and consequently H_2_O_2_)-dependent and peroxynitrite-dependent oxidation of H_2_DCF. Notably, EBS prevented H_2_DCF oxidation in cells exposed to NO^•^ and peroxynitrite in the presence of SIH as well, indicating that the oxidation of H_2_DCF is peroxynitrite-dependent regardless of a chelator’s presence (Figure 3). In other words, the SIH does not introduce different mechanisms of H_2_DCF oxidation. 

### 3.4. The Relative Enhancement Effect of LIP Chelation on Peroxynitrite-Dependent H_2_DCF Oxidation

A dimensionless kinetic parameter (q) was employed to quantify the relative enhancement effect of LIP chelation on peroxynitrite-dependent H_2_DCF oxidation for all the selected cell types in the study. This parameter (Table 1) was calculated as the ratio of the rate of DCF fluorescence increase in the absence and presence of the chelator SIH, using the data presented in Figure 3, bottom panels. The assumption was made that cells exhibit similar behavior in the absence and presence of the chelator, except for the inhibition of the peroxynitrite and LIP reaction. Table 1 also presents the LIP_T_ concentration for each cell type (Figure 1).

### 3.5. LIP Does Not React with Peroxynitrite-Derived Radicals in Cells

**Figure 4:**
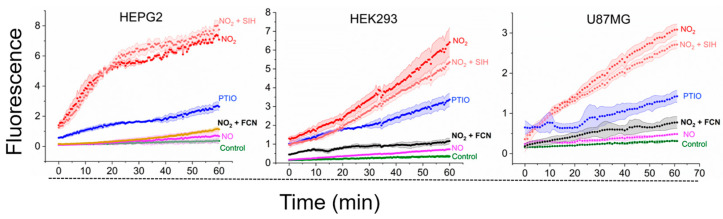
${\mathrm{NO}}_{2}^{•}$-dependent intracellular oxidation of H_2_DCF and the effects of SIH. DCF fluorescence traces of HEPG2 (left), HEK 293 (center), and U87-MG (right) cells exposed to ${\mathrm{NO}}_{2}^{•}$ fluxes. NO^•^: DETA/NO (250 μM); PTIO: PTIO (250 μM); ${\mathrm{NO}}_{2}^{•}$: DETA/NO (250 μM) and PTIO (250 μM); ${\mathrm{NO}}_{2}^{•}$ + SIH: DETA/NO (250 μM), PTIO (250 μM), and SIH (100 µM); ${\mathrm{NO}}_{2}^{•}$ + FCN: DETA/NO (250 μM), PTIO (250 μM), and FCN (1 mM). Briefly, a suspension of H_2_DCF-loaded cells was transferred to 96-well plates (12 × 10^6^ cells/mL) and treated with the combination of DETA/NO and PTIO plus or minus SIH or FCN, and the fluorescence data acquisition was registered every minute for one hour. The data represent the mean ± S.D of four experiments. The assays were performed in PBS containing DTPA (100 μM) at pH 7.4 and 37 °C using a multiple well plate reader with the following fluorescence acquisition parameters: λex = 498 nm, λem = 523 nm, and ex and em slit width = 9 and 15 nm, respectively.

We used ${\mathrm{NO}}_{2}^{•}$ as the prototype to investigate whether peroxynitrite or its derived radicals reacted with the LIP in cells. The ${\mathrm{NO}}_{2}^{•}$ radical is known for its rapid free diffusion into the cytosolic space [46] and high reactivity with H_2_DCF (*k* = 1.3 × 10^7^ M^−1^s^−1^). A flux of the ${\mathrm{NO}}_{2}^{•}$ radical was achieved by combining the NO^•^ donor DETA/NO with an excess of 2-phenyl-4,4,5,5-tetramethylimidazoline-1-oxyl 3-oxide radical (PTIO), which oxidizes NO^•^ to ${\mathrm{NO}}_{2}^{•}$ extracellularly (PTIO is cell membrane-impermeable) [47]. In optimized conditions previously established [28], 250 µM PTIO provided a constant flux of ${\mathrm{NO}}_{2}^{•}$ devoid of NO^•^ and other oxidants [28]. H_2_DCF-loaded cells were placed in 96-well plates and exposed to DETA/NO in the presence of PTIO, with and without the LIP chelator SIH, along with different control runs (Figure 4). The PTIO significantly increased DCF formation compared to the NO^•^ donor alone, consistent with ${\mathrm{NO}}_{2}^{•}$ formation and ${\mathrm{NO}}_{2}^{•}$-dependent H_2_DCF oxidation intracellularly. To confirm that such oxidation was ${\mathrm{NO}}_{2}^{•}$-dependent, control experiments in the presence of the well-known ${\mathrm{NO}}_{2}^{•}$ scavenger FCN were conducted. FCN fully prevented the oxidation of H_2_DCF to DCF in the presence of the NO^•^ donor and PTIO, likely by reducing ${\mathrm{NO}}_{2}^{•}$ to ${\mathrm{NO}}_{2}^{-}$ extracellularly. These results confirmed that H_2_DCF efficiently reacts with ${\mathrm{NO}}_{2}^{•}$ in HEPG2, HEK293, and U87-MG cells, effectively competing for this oxidant with cellular targets under the experimental conditions.

More importantly, the LIP chelator SIH showed no impact on the ${\mathrm{NO}}_{2}^{•}$-dependent oxidation of H_2_DCF in all cell types tested (compare the red shaded traces; Figure 4). This consistency, similar to observations in RAW 264.7 cells, led to relevant conclusions. First, the lack of an effect on ${\mathrm{NO}}_{2}^{•}$-dependent oxidation by the LIP chelator indicates that the LIP does not directly react with ${\mathrm{NO}}_{2}^{•}$ in cells. While the LIP, when isolated, probably reacts with ${\mathrm{NO}}_{2}^{•}$, its low sub- to low micromolar concentrations render it unable to compete with multiple abundant cellular targets of ${\mathrm{NO}}_{2}^{•}$. This conclusion was then extrapolated to the other peroxynitrite-derived reactive species like OH^•^ and ${\mathrm{CO}}_{3}^{\bar{•}}$, suggesting that the LIP likely acts upstream of peroxynitrite-derived radical oxidants. Specifically, the LIP reacts with peroxynitrite. Second, the observation that SIH did not affect the rate of H_2_DCF oxidation in cells exposed to NO^•^ and PTIO dismisses alternative hypotheses concerning the origin of the H_2_DCF oxidant and the effects of SIH in cells exposed to DMNQ/NO^•^. These alternatives include the slow reaction of NO^•^ with O_2_ (which produces ${\mathrm{NO}}_{2}^{•}$ [48]) and artifact production of $O_{2}^{\bar{•}}$ by the putative intermediate H_2_DCF-derived DCFH^•^ radical. If these were relevant, SIH would similarly enhance ${\mathrm{NO}}_{2}^{•}$-dependent H_2_DCF oxidation.

## 4. Discussion

The SIH chelator consistently increased peroxynitrite-dependent H_2_DCF oxidation in all cell types tested so far. The classic iron chelator 2,2-bipyridine also elicited a chelator effect (consistent to our previous report [27]), showing that this effect is not specific to SIH, but actually a general property of membrane-permeable LIP chelators. Impermeable chelators have no effect [27]. Moreover, the enhancement effect of SIH on H_2_DCF oxidation is cell-dependent, as there was no effect by chelators on peroxynitrite-dependent H_2_DCF oxidation in cell-free systems [27]. We endorse the proposition that the LIP reacts with peroxynitrite, partially preventing its reaction with CO_2_, and consequently reducing the concentration of peroxynitrite and its derived radicals. However, alternative hypotheses exist.

One such possibility is the interference of SIH on H_2_DCF properties. However, spectroscopic data did not indicate that aqueous iron (II) and SIH, alone or in combination, interacted with H_2_DCF or DCF to alter their absorbance and fluorescence properties, or acted as an internal filter [27]. Another explanation for the chelator effect is that free SIH or the LIP/SIH complex generates $O_{2}^{\bar{•}}$ or other potential oxidants [22,23]. However, neither the free SIH nor its respective iron(II) complex consumed oxygen or produced $O_{2}^{\bar{•}}$ in cell-free assays (please see the supplementary information of our previous publication [27]). Furthermore, there was no discernible $O_{2}^{\bar{•}}$- or H_2_O_2_-dependent H_2_DCF oxidation in the presence of DMNQ relative to controls, and this was not affected by SIH or EBS (Figure 3, top three panels). In addition, to refute the hypothesis that SIH produces oxidants, these results indicate that the chemistry of H_2_O_2_ with the LIP or iron peroxidases is not pertinent to the intracellular oxidation of H_2_DCF under the study conditions [23]. Another explanation is that SIH increased the NO^•^ availability, but the steady-state NO^•^ concentration was not affected by SIH (please see the supplementary information of our previous publication [27]). The LIP reacts with NO^•^ to form DNIC, suggesting that the increased oxidation of H_2_DCF in the presence of SIH might be due to DNIC. However, the iron in cellular DNIC is not chelatable [21], and the SIH effect remained essentially unchanged whether it was added before or during the experiments involving the exposure of cells to peroxynitrite [28], arguing against any peroxynitrite antioxidant role of DNIC under our experimental conditions. Finally, it is unreasonable to assume that SIH inhibits or decreases the concentrations of cellular antioxidants that prevent peroxynitrite formation (e.g., SODs) or could potentially compete with H_2_DCF for peroxynitrite-derived radicals (e.g., GSH) within the timeframe of the experiments. Therefore, the most plausible explanation for the observed results is that SIH binds to the LIP, preventing the reaction between the LIP and peroxynitrite, thereby attenuating the oxidation of the fluorescent indicator (Scheme 1). When this reaction is prevented by chelators, oxidation increases.

It is noteworthy that the chelator effect was evident with activated macrophages producing endogenous NO^•^ [27]. In addition, the LIP and peroxynitrite reaction might also protect biological targets from peroxynitrite-induced oxidation, as indicated by the increased content of carbonylated proteins in macrophage cells exposed to peroxynitrite fluxes in the presence of SIH [27]. Accordingly, as elaborated before [27], we have identified a few earlier studies in the literature indicating that chelators enhance potential peroxynitrite-dependent oxidation or produce biological effects [49,50,51]. While the authors proposed alternative explanations, their findings align with the hypothesis that the LIP reacts with peroxynitrite. The observation that the most abundant TPs (Peroxiredoxin 1 and 2) in human neutrophils remain locked in the inactive oxidized disulfide state is intriguing [52] and may be crucial for their function, avoiding wasteful degradation of peroxynitrite and the oxidants that neutrophils themselves may produce during their active immune response. Investigating the peroxynitrite-reductase activity of the LIP in active neutrophils is of significant interest and might provide valuable insights into the potential role of the LIP as a self-defense mechanism, aiding neutrophils in sustaining a prolonged immune response during inflammatory conditions.

The kinetic parameter q that represents the antioxidant effect of the LIP ranges from 0.54 to 0.67 across various cell types (Table 1). This suggests that the LIP consistently attenuates peroxynitrite-dependent H_2_DCF oxidation by approximately 30–40%, despite a tenfold variation in LIP concentration (Table 1). This is not totally surprising, since the antioxidant activity of the LIP against peroxynitrite reflects not only the LIP concentration and reactivity with peroxynitrite, but also the competition between the LIP and other key targets of peroxynitrite in cells, such as CO_2_ and TPs (Scheme 1). Specifically, the TPs are potent peroxynitrite reductase enzymes [53,54,55], and their expression and activities can vary among different cell types, potentially influencing the overall dynamics of peroxynitrite-dependent reactions and the effect of the LIP. This is particularly notable under our experimental conditions, given the inherent production of their main substrate, H_2_O_2_, through DMNQ-derived $O_{2}^{\bar{•}}$ disproportionation.

The precise identity of the LIP remains elusive. This uncertainty prevents direct studies of the LIP’s interactions with peroxynitrite and other oxidants and ligands like NO^•^. Based on available thermodynamic constants of Glutathione (GSH) and Fe(II) binding [17,56,57], GSH emerges as a potential candidate capable of binding the LIP in cells. Notably, the model LIP complex [Fe(GS)(H_2_O)], generated from an aqueous mixture of Fe(II) and excess GSH, has been demonstrated to increase peroxynitrite consumption relative to that of spontaneous acid-catalyzed peroxynitrite decomposition and its direct reaction with GSH [27]. This observation suggests that [Fe(GS)(H_2_O)] can indeed react with peroxynitrite. The other possibility is that the LIP is protein-bound, which is grounded in different experimental evidence. For example, the LIP reacts with NO^•^, quantitatively yielding macromolecule-bound DNIC [21]. Thus, in this sense, the protein-bound LIP reacts with NO^•^, yielding protein-bound DNIC. Also, our recent observation that the cytosolic LIP binds tightly (Kd ≅ 10^−2^ µM) to cellular constituents [20] aligns with the documented binding strength of chelatable Fe(II) in cytosolic mononuclear non-heme enzymes such as prolyl hydroxylases (PHDs) [58,59]. Additionally, poly(rC)-binding protein 1 (PCBP1) in association with GSH has been proposed to weakly bind the cytosolic LIP and to act as an iron chaperone [16]. Unfortunately, the reaction of these Fe-proteins with peroxynitrite remains unexplored. Future investigations delving into the interactions between these Fe-proteins and peroxynitrite could offer valuable insights into the identity of the LIP and the mechanisms, kinetics, and products of the reaction of the LIP with peroxynitrite and other species.

The specifics of the reaction between the LIP and peroxynitrite, including the mechanism and resulting products, remain elusive. A proposed hypothesis involves the LIP reducing peroxynitrite to ${\mathrm{NO}}_{2}^{-}$ (Scheme 1), inspired by analogous reactions of peroxynitrite with divalent metal complexes [60] and hemeproteins. Studies on ferrous hemeproteins such as myeloperoxidase [61], deoxymyoglobin, and deoxyhemoglobin [62] highlight their ability to reduce peroxynitrite to ${\mathrm{NO}}_{2}^{-}$ with rate constants (k ≥ 10^6^ M^−1^s^−1^) notably larger than those of peroxynitrite’s reaction with CO_2_. This hypothesis predicts the production of the oxidant oxy-ferryl species LIP-Fe=O^4+^, introducing a degree of uncertainty regarding the potential anti-oxidant role of the LIP against peroxynitrite. Despite this, it has to be emphasized that LIP-Fe=O^4+^ is probably less reactive than peroxynitrite and peroxynitrite-derived radical oxidants. LIP-Fe=O^4+^ is analogous to hemeperoxidase compound II, the reaction of which typically represents the rate limiting step in the one-electron catalytic cycle of various hemeperoxidases with most one-electron reducing agents (such as ${\mathrm{NO}}_{2}^{-}$ [63], NO^•^ [64]). Specifically, in the oxidation of H_2_DCF by the horseradish peroxidase (HRP)/H_2_O_2_ system, HRP-Compound II is the intermediate that accumulates and can be detected spectrophotometrically [65]. The oxy-ferryl species LIP-Fe=O^4+^ may be reduced by H_2_DCF, but in our experimental setup and in physiological conditions, LIP-Fe=O^4+^ might be more efficiently neutralized by abundant cellular reducing agents like GSH, which are present in mM concentrations. The role of GSH in two existing hypotheses of the LIP identity [16,17] suggests that GSH, in close proximity to oxyferryl species, may serve as a sacrificial reductant. Accordingly, depletion of GSH has been observed to increase peroxynitrite-dependent oxidation, although it has to be acknowledged that this effect might not be distinctly separated from the direct scavenging of peroxynitrite-derived oxidants by GSH. The reduction of LIP-Fe=O^4+^ to the ferrous LIP would make the LIP a peroxynitrite reductase system. Consistently, accumulated data and results from our previous [27,28] and current studies (Figure 3) show that the rate of H_2_DCF oxidation in the absence of SIH is constant, suggesting that the concentration of the LIP fraction reactive toward peroxynitrite does not appreciably decrease during the experimentally observed time window. In RAW 264.7 cells, the cumulative production of peroxynitrite significantly surpassed the concentration of the LIP in 60 min runs [28], providing support for the hypothesis that the LIP catalytically removes peroxynitrite. Using a simplified LIP and peroxynitrite reaction model and the dimensionless kinetic parameter q (Table 1), we estimated that the rate constant for the hypothetical reaction between the LIP and peroxynitrite falls within the range of 1–40 × 10^6^ M^−1^s^−1^ in RAW 264.7 cells [28]. The kinetic parameter q, derived from the same model, yielded rate constants for the LIP and peroxynitrite reaction within the same range for all cell types investigated in this study. The catalytic characteristics and the high estimated rate constant further support the notion that the LIP’s role in removing peroxynitrite is a significant and efficient process. 

Together, the consistent LIP binding properties [20] and reactivity toward peroxynitrite in different cell types imply a similar molecular nature of cellular LIP ligands across various cell types. This consistency aligns with its role as a ubiquitous cellular iron source that is involved in critical processes, such as the metalation of nascent apo-iron proteins and ferroptosis. Furthermore, the reaction between the LIP and peroxynitrite may potentially impact cellular iron homeostasis and ferroptosis by influencing the redox state, binding properties, and reactivity of the LIP.

## 5. Conclusions

The study reveals that the reaction between the LIP and peroxynitrite is widespread, rapid, and potentially catalytic in diverse cell types. This suggests that the LIP could serve as a ubiquitous antioxidant system against peroxynitrite, offering complementary or substitutional protection to cells, especially in conditions in which peroxynitrite production is locally elevated, such as in infections and inflammation.

## Figures and Tables

**Table 1:** The effect of the LIP on the rate of peroxynitrite-dependent oxidation of H_2_DCF in cells.

Cell Type	HEPG2	KEK293	U87-MG	RAW 264.7
LIP_T_ (µM)	0.26 ± 0.03	0.7 ± 0.1	0.53 ± 0.03	1.9 ± 0.4 *
q ^#^	0.60	0.54	0.67	0.62 *

^#^ q is determined by dividing the rate of DCF fluorescence increase in cells exposed to DMNQ/NO^•^ in the absence of SIH by the rate of DCF fluorescence increase in the presence of SIH using the data shown in Figure 3. * Ref. [28].

## Data Availability

Data are contained within the article.

## Abbreviations

GSH, Glutathione; DCF, 2′,7′-dichlorofluorescein; H_2_DCF-DA, 2′,7′-dichlorodihydrofluorescein diacetate; DNIC, dinitrosyl iron complex; DTPA, diethylenetriaminepentaacetic acid; EBS, 2-Phenyl-1,2-benzisoselenazol-3(2H)-one; BOR, boronates; CBA, coumarin-7-boronic acid; COH, 7-hydroxy coumarin; FCN, sodium hexacyanoferrate (II); LIP, labile iron pool; SIH, salycylaldehyde isonicotinoyl hydrazone; DETA/NO, 2,2′-(Hydroxynitrosohydrazono)bis-ethanimine; PTIO, 2-Phenyl-4,4,5,5-tetramethylimidazoline-1-oxyl 3-oxide; Calcein-AM, Glycine,N,N′-[[3′,6′-bis(acetyloxy)-3-oxospiro[isobenzofuran-1(3H),9′-[9H]xanthene]-4′,5′-diyl]bis(methylene)]bis[N-[2-[(acetyloxy)methyoxy]-2-oxoethyl]-, bis[(acetyloxy)methyl] ester; Calcein (CA), 2,2′,2″,2‴-(((3′,6′-Dihydroxy-3-oxo-3H-spiro[isobenzofuran-1,9′-xanthene]-2′,7′-diyl)bis(methylene))bis(azanetriyl))tetraacetic acid; DMNQ, 2,3-dimethoxy-1,4-naphthalenedione; HRP, horseradish peroxidase.

## Appendix A. Justification for the Choice of Salycylaldehyde Isonicotinoyl Hydrazone (SIH) as the LIP Chelator

SIH is a cell membrane-permeable iron chelator that accesses the cytosolic space of cells and binds the LIP within minutes, forming the [Fe(SIH)_2_] complex [36]. Importantly, this complex does not engage in redox cycling to produce oxidants. In fact, SIH has previously demonstrated its efficacy in attenuating iron-dependent oxidative stress-induced mitochondrial injury and cell death [66]. Moreover, in an aqueous solution, the [Fe(SIH)_2_] complex does not directly react with peroxynitrite [27]. Given that peroxynitrite redox reactions occur through the inner-sphere mechanism [67], which requires binding to the substrate, SIH’s ability to avoid peroxynitrite binding to transition metal ions explains its potential in preventing metal oxidation by peroxynitrite. Additionally, neither free SIH nor the [Fe(SIH)_2_] complex interferes with the absorbance and fluorescence properties of DCF [27].

## Appendix B. Justification for the Use of H2DCF

The use of H_2_DCF is subject to controversy, primarily for two reasons. Firstly, criticism arises from the fact that H_2_DCF does not directly react with peroxynitrite. As rationalized below, we argue that this characteristic can actually be advantageous. Some critics may advocate for the use of fluorescent boronate compounds as better indicators due to their direct and high-rate constant reaction with peroxynitrite. However, using boronate compounds presents challenges, as outlined previously [27,28]. High concentrations of boronate can outcompete the LIP for peroxynitrite, making it difficult to observe the chelator’s effect on boronate oxidation. Thus, low concentrations of boronate would be more appropriate, but that poses another issue. In this scenario, only a fraction of available peroxynitrite reacts with boronate (which limits the sensitivity); thus, in the presence of an LIP chelator, most peroxynitrite that would otherwise react with the LIP might not react with boronate, but rather with other cellular constituents (such as TPs and CO_2_, Scheme 1). Consequently, inhibiting the LIP-peroxynitrite reaction with chelators does not yield experimentally detectable increases in peroxynitrite-dependent boronate oxidation in either scenario of high or low concentrations of boronate. Boronate compounds are excellent for detecting peroxynitrite [68], but our study aims to investigate the hypothetical reaction between peroxynitrite and the LIP, not merely to detect peroxynitrite. Therefore, the use of an indirect peroxynitrite indicator like H_2_DCF, along with proper control experiments, aligns better with the objectives and requirements of the research. A second notable criticism of using H_2_DCF pertains to the complexity of the DCF formation mechanism. This is a multiple step process, supposedly starting with the oxidation of H_2_DCF to the putative radical DCFH^•^ [69]. This initial oxidation step requires strong one-electron oxidants, such as radicals derived from peroxynitrite or high-valent oxy-ferryl species resulting from reactions of peroxides (e.g., H_2_O_2_) with heme proteins and heme peroxidases [41,44]. The subsequent fate of DCFH^•^ involves dismutation, or more likely reaction with O_2_, yielding fluorescent DCF and $O_{2}^{\bar{•}}$ in the process [69]. This raises concerns, as the artefactual $O_{2}^{\bar{•}}$ produced in this pathway could potentially react with NO^•^ and stimulate peroxynitrite-dependent oxidation of H_2_DCF under certain conditions. However, it is crucial to note that in our experimental design, we used DMNQ to deliberately generate $O_{2}^{\bar{•}}$. This is significant because the $O_{2}^{\bar{•}}$ (and consequently H_2_O_2_) derived from the DCFH^•^ pathway is arguably negligible compared to the $O_{2}^{\bar{•}}$ derived from DMNQ itself. By employing DMNQ, we ensured that the $O_{2}^{\bar{•}}$ generated in our experimental conditions was predominantly derived from DMNQ. Moreover, it is not clear how the LIP chelator SIH would increase DCF formation by this mechanism.
